# Nutritional, Bioactive, Antioxidant, and Safety Assessment of Some Wild Edible Medicinal Plants Commonly Consumed in Morocco

**DOI:** 10.1155/ijfo/5125567

**Published:** 2026-04-24

**Authors:** Abdelghani Aboukhalaf, Youssef Lahlou, Houda El Yacoubi, Atmane Rochdi, Adil Kalili, Jamila El Biyad, Rekia Belahsen

**Affiliations:** ^1^ Department of Biology, Laboratory of Natural Resources and Sustainable Development, Faculty of Sciences, Ibn Tofail University, Kenitra, Morocco, uit.ac.ma; ^2^ Department of Biology, Faculty of Sciences, Chouaib Doukkali University, El Jadida, Morocco, ucd.ac.ma

**Keywords:** acute toxicity, antioxidant compounds, nutritional profile, wild edible medicinal plants

## Abstract

Despite the widespread use of wild edible medicinal plants (WEMPs) in Morocco, information on their nutritional value, bioactive composition, and safety aspects is limited. Thus, the present study was aimed at evaluating the nutritional value (proximate and mineral contents), phytochemical properties, and acute toxicity of *Dysphania ambrosioides*, *Ziziphus lotus*, and *Origanum vulgare* collected from central Morocco. Nutritional composition was determined using standard food analysis methods, while total phenolic and flavonoid contents were quantified colorimetrically. Antioxidant activities were assessed by DPPH, ABTS, and FRAP assays. The acute safety profile of the plant extracts was evaluated through acute toxicity testing in rats. Results revealed that the studied WEMPs contained valuable nutrients in the respective ranges of moisture (6.25%–10.23%), ash (4.81%–14.85%), protein (6.98%–20.43%), fat (2.26%–2.88%), carbohydrates (63.75%–85.33%), and energy (345.45–395.16 kcal·100 g^−1^). They were also rich in essential minerals, particularly magnesium (46.76–95.77 mg·100 g^−1^), iron (1.73–4.31 mg·100 g^−1^), and manganese (1.65–2.02 mg·100 g^−1^). The total phenolic and flavonoid contents ranged from 64.45 to 207.30 mg GAE·g^−1^ extract and from 18.84 to 117.18 mg QE·g^−1^ extract, respectively. Antioxidant assays showed strong correlations with phenolic and flavonoid contents. Acute toxicity results indicated that all extracts were well tolerated, with LD50 values higher than 5000 mg·kg^−1^ body weight, suggesting the absence of acute toxicity under the experimental conditions. Overall, the findings demonstrate that these WEMPs represent valuable sources of nutrients and bioactive compounds with antioxidant potential and could serve as natural dietary antioxidants to help mitigate oxidative stress.

## 1. Introduction

Food insecurity and malnutrition are among the most critical global challenges of our time [1]. Approximately one in seven people worldwide suffer from nutrient deficiencies, which increases their susceptibility to illness [2], thereby representing a major impediment to fulfilling the Sustainable Development Goals related to poverty eradication (SDG1), food security (SDG2), and human health (SDG3) [3]. The limited availability and unstable supply of nutrient‐dense foods have prompted increasing interest in identifying affordable and alternative nutritional sources. In this context, wild edible medicinal plants (WEMPs) constitute valuable resources provided by nature and represent promising alternatives to combat food insecurity and health issues [4]. Defined as “plant resources that grow under natural conditions (wild) and are harvested or collected for human consumption and used as food, dietary supplements, and medical treatment” [2], WEMPs contribute significantly to the diets of rural populations by supplying essential nutrients, minerals, and a wide range of bioactive phytochemicals [5]. Their capacity to thrive under drought, pest pressure, and poor soil conditions further enhances their relevance in the face of current climatic and agricultural constraints [5].

Morocco, located at the crossroads of the Mediterranean, Atlantic, and Saharan biogeographic zones, is recognized for its exceptional plant biodiversity, with more than 5211 recorded vascular plant species of which approximately 17% are endemic, placing the country among the Mediterranean regions with the greatest plant diversity [6]. Morocco′s diverse flora offers a wealth of wild and semi‐WEMPs that can be used for food or medicinal purposes. Recent investigations have begun to characterize the nutritional and bioactive profiles of Moroccan WEMPs. For example, Ibourki et al. [7] reported that 20 wild medicinal and aromatic plants exhibited high mineral levels often exceeding those of cultivated species and a low lipid content. Complementary findings by Ghanimi et al. [8] indicated that *Emex spinosa*, *Silene vulgaris*, and *Malva sylvestris* were particularly rich in carbohydrates, ash, and proteins, confirming the nutritional potential of several Moroccan wild species. Mineral variability has also been documented with Essaih et al. [9] reporting very high levels of potassium (K), calcium (Ca), and iron (Fe) in several species.

Beyond nutritional composition, several studies have highlighted the biological potential of Moroccan WEMPs. Aboukhalaf et al. [10] reported notable antimicrobial activity in species such as *Foeniculum vulgare*, *Papaver rhoeas*, *Rubia peregrine*, *Ziziphus lotus*, *Origanum vulgare*, and *Mercurialis annua*. Further work by Aboukhalaf et al. [11] showed that *Scolymus hispanicus* displays strong antioxidant and antibacterial activities.

Although these studies provide valuable insights, current knowledge remains fragmented. Most available investigations examine either nutritional composition, antimicrobial activity, or selected phytochemical markers in isolation. Comprehensive evaluations integrating proximate composition, bioactive compounds, antioxidant capacity, and toxicological assessment are still scarce or entirely absent for many widely consumed Moroccan species. Moreover, standardized and comparable datasets covering multiple biochemical and biological parameters remain limited.

To bridge this knowledge gap, the present study offers a holistic assessment of selected Moroccan WEMPs by examining their proximate and mineral compositions, total phenolic and flavonoid contents, antioxidant activity, and acute toxicity. By generating integrated and standardized data for species that are regularly consumed but insufficiently documented, this work contributes to strengthening the scientific basis for their nutritionally responsible and sustainable use.

## 2. Materials and Methods

### 2.1. Collection of Plant Material

The WEMPs selected for this study were collected in March 2023 from various locations in Sidi Bennour, Morocco. The plant species were taxonomically identified and authenticated by a botanist at the herbarium of Chouaïb Doukkali University. Relevant information on the investigated plants is provided in Table 1.

**Table 1:** List of WEMPs commonly consumed by the Moroccan population.

Scientific name	Botanical family	Local name	Edible parts	Food use	Medicinal use	References
*Dysphania ambrosioides* (L.) Mosyakin & Clemants (MZ5)	Amaranthaceae	*Mkhinza*	Leaves	Vegetables, aroma	Fever, anthelmintic, antidiarrheal, colds, detersive, influenza	[12–14]
*Ziziphus lotus* (L.) Lam (NG3)	Rhamnaceae	*Nbeg*/*Sedra*	Fruits	Snack	Anthelmintic, wound healing, urinary tract infections	[13]
*Origanum vulgare* L. (ZT6)	Lamiaceae	*Zaatar*	Leaves	Aroma, spices, drink	Cold, antiseptic, diarrhea, influenza, cough, intestinal parasites	[13, 14]

### 2.2. Preparation of Crude Extracts

Plant materials were first rinsed thoroughly with distilled water and then dried in the open air. Once completely dried, the samples were ground to a fine powder. A 100‐g portion of each powdered sample was extracted by maceration in methanol using a solid‐to‐liquid ratio of 1:10 (*m*/*v*) and shaken for 24 h at room temperature (approximately 20°C–27°C) using a shaker machine (LMMRS‐110, United States). The procedure was repeated three times to ensure complete extraction. Previous studies have reported that methanol is one of the most effective solvents for extracting plant compounds, as it provides the highest extraction efficiency [5, 15]. The resulting macerate was passed through Whatman No. 1 filter paper to separate the solid residues from the liquid phase. The filtrate was then concentrated under reduced pressure using a vacuum evaporator (RE100‐Pro, DLAB, China) to obtain the crude extract, which was stored at 4°C until further analysis. The extraction yield was determined by applying the following formula:

Yield %=mass of dried extractmass of dry plant material×100.

### 2.3. Animals

Healthy, nonpregnant young (8–12 weeks) female Wistar rats (140–160 g) were sourced from the Animal House of the Department of Biology, Faculty of Sciences, Ibn Tofail University (Morocco), and used in the present study. Female rats were used in accordance with OECD guidelines, which recommend their use in acute toxicity testing due to their generally higher sensitivity to toxic substances. The animals were housed in standard cages and acclimatized for 7–9 days under controlled environmental conditions (23^°^C ± 3^°^C, 12‐h light/dark cycle) prior to the initiation of treatment. Food and water were supplied freely throughout the acclimatization period.

### 2.4. Nutritional Value

#### 2.4.1. Basic Composition

Most components of the basic composition, namely, moisture, protein, fat, and ash, were analyzed following standardized methodologies established by the AOAC [16]. Moisture content was measured by oven‐drying the samples at 100^°^C ± 5^°^C for 5–6 h until a stable weight was achieved (AOAC 930.04). Crude protein was measured by applying the Kjeldahl technique, with nitrogen values converted to protein using the factor of 6.25 (AOAC 984.13). Lipid content was quantified gravimetrically after continuous extraction with hexane (60°C–80°C) in a Soxhlet apparatus for approximately 6–8 h (AOAC 920.39). Ash determination was performed by combusting the plant material in a muffle furnace at 550°C for 5–6 h (AOAC 942.05). Total carbohydrate content was calculated by difference on a dry weight basis (Equation 1):

(1) Carbohydrates%=100–protein+fat+ash.

The energy value (kcal) was estimated using the Atwater conversion factors (Equation 2):

(2) Energykcal=proteins%×494+lipids%×+carbohydrates%×.

#### 2.4.2. Macro‐ and Microelement Analysis

##### 2.4.2.1. Sample Digestion.

Mineral analyses were performed following the protocol described by Ait Bouzid et al. [17], with slight modifications. Dried leaf samples (0.5 g) were digested using a temperature‐controlled digestion system (Memmert, Schwabach, Germany). Digestion was carried out in 8 mL of concentrated nitric acid (HNO_3_, 65%) and 2 mL of hydrogen peroxide (H_2_O_2_, 30% *w*/*v*) according to the following program: (i) 5 min to reach 160°C, (ii) 5 min at 160°C, (iii) 3 min to reach 210°C, and (iv) 10 min at 210°C. After cooling, the digested solutions were diluted to a final volume of 25 mL with ultrapure water.

##### 2.4.2.2. Inductively Coupled Plasma Optical Emission Spectrometry (ICP‐OES) Determination.

The concentrations of selected macroelements and microelements (Ca, sodium [Na], K, magnesium [Mg], Fe, copper [Cu], manganese [Mn], and zinc [Zn]) were determined using ICP‐OES (PerkinElmer, Waltham, United States), equipped with an autosampler (ASX‐520, Teledyne CETAC Technologies, Omaha, United States) and a charge‐coupled device (CCD) detector. The ICP‐OES operating conditions were as follows: plasma gas flow rate: 14 L·min^−1^; auxiliary gas flow rate: 0.2 L·min^−1^; nebulizer gas flow rate: 0.8 L·min^−1^; RF power: 1300 W; sample flow rate: 1.3 mL·min^−1^; flush time: 7 s; viewing mode: axial. All measurements were performed in triplicate.

##### 2.4.2.3. Quality Control Procedure.

Calibration curves for all analyzed elements showed coefficients of determination (*R* ^2^) higher than 0.995. An internal quality control solution was analyzed at the beginning, middle, and end of each analytical batch to monitor instrument stability. Method accuracy and precision were evaluated using the certified reference material GBW 07605 (National Research Center for Certified Reference Materials, Langfang, China).

The limits of detection (LOD) and quantification (LOQ) were calculated using Equations (3) and (4):

(3) LOD=Xb13.31+Sb,

(4) LOQ=Xb1101+ Sb,

where Xb1 represents the mean concentration of the blank and Sb1 denotes its standard deviation. The analytical wavelengths, LODs, LOQs, coefficients of determination (*R* ^2^), and recovery percentages for each element are reported in Table 2.

**Table 2:** ICP‐OES analytical parameters and validation data for mineral element determination.

Elements	Wavelengths (nm)	LOD (mg·L^−1^)	LOQ (mg·L^−1^)	Coefficient of determination (*R* ^2^)	Recovery (%)
Mg	285.213	0.003	0.005	0.999	107.44
Ca	317.933	0.03	0.08	0.999	101.18
Na	589.592	0.30	1.0	0.999	95.45
K	766.490	0.03	0.05	0.999	95.36
Zn	213.857	0.016	0.05	0.999	106.61
Fe	239.562	0.02	0.05	0.999	91.28
Mn	257.610	0.0026	0.008	0.999	103.34
Cu	324.752	0.001	0.003	0.999	91.96

### 2.5. Quantification of Total Phenolic Content (TPC)

Quantification of TPC of the plant extracts was carried out according to the protocol of Folin–Ciocalteu as stated by Kim et al. [18]. The absorbance of phenolic compounds was measured at 760 nm using a spectrophotometer (Jenway 6300, United States). A calibration curve was constructed using gallic acid, and the regression equation (*Y* = 0.0042*x* + 0.0393; *R* ^2^ = 0.9943, Figure S1) was used to calculate the TPC of different extracts. The results were expressed both as milligrams gallic acid equivalents per gram of extract (mg GAE·g^−1^ extract) and per gram of dry plant material (mg GAE·g^−1^ DW).

### 2.6. Quantification of Total Flavonoid Content (TFC)

The aluminum chloride method was used to assess the TFC in the plant extracts using protocol [18]. The absorbance of flavonoid content was measured at 510 nm. The calibration curve was plotted using quercetin, and the regression equation (*Y* = 0.0009*x* + 0.009; *R* ^2^ = 0.9828, Figure S2) was used to calculate the TFC of different extracts. The concentrations were expressed both as milligram quercetin equivalent per gram of extract (mg QE·g^−1^ extract) and per gram of dry plant material (mg QE·g^−1^ DW).

### 2.7. Antioxidant Activity

In vitro antioxidant activity of the selected WEMPs was assessed using three different assays, including DPPH (2,2‐diphenyl‐1‐picrylhydrazyl), ABTS, and FRAP (ferric reducing antioxidant power).

#### 2.7.1. Determination of Antioxidant Activity by DPPH Assay

The free radical inhibitory potential of each plant extract was evaluated based on its capacity to scavenge the DPPH free radical, following a previously reported method [19]. A methanolic DPPH solution (0.004%, *w*/*v*) was mixed with the extract samples at a 1:1 (*v*/*v*) ratio and thoroughly shaken. The mixtures were incubated in the dark at room temperature for 30 min, after which absorbance was measured at 517 nm using a UV‐visible spectrophotometer. The experiment was conducted in triplicate, with data expressed as IC_50_ values (the concentration in mg/mL at which DPPH radicals were scavenged by 50%), which were estimated by linear regression analysis of the dose–response curve obtained by plotting inhibition percentage against concentration (Figure S3).

#### 2.7.2. Determination of Antioxidant Activity by FRAP Assay

The FRAP assay of each extract was carried out according to minor modified methods of Oh et al. [20]. The FRAP reagent was prepared by mixing 300 mM acetate buffer (pH 3.6), 10 mM TPTZ, and 20 mM FeCl_3_·6H_2_O solutions in a 10:1:1 ratio. For the assay, a reaction mixture was prepared by combining 1.5 mL of the FRAP reagent with 50 *μ*L of the plant extracts or ascorbic acid (using as a standard) and 150 *μ*L of distilled water. The mixture was incubated at 37°C for 20 min, and absorbance was subsequently measured at 593 nm. Results were expressed as IC_50_ values, which give an absorbance of 0.5 for reducing power, and were calculated by linear regression analysis of the dose–response curve obtained by plotting absorbance against extract concentration (Figure S4). All tests were performed in triplicate.

#### 2.7.3. Determination of Antioxidant Activity by ABTS Radical Scavenging Assay

The ABTS assay was performed by a modification of the method previously described by Oh et al. [20], according to the procedure below:

− Preparation of the radical solution: ABTS solution (7 mM) and potassium persulfate solution (2.45 mM) were mixed (2:1) and incubated in the dark for 16 h.
− The ABTS mixture: The radical solution of ABTS was diluted with ethanol at a ratio of 1:60 to reach an absorbance of 0.700 at 734 nm.
− The assay: Mixing 50 *μ*L of various concentrations of the plant extracts or ascorbic acid (500, 250, 125, 62.5, 31.25, and 15.62 *μ*g·mL^−1^, prepared from stock solutions of 1 mg·mL^−1^) with 1 mL of the diluted ABTS solution.
− Incubation of the mixture in darkness for 15 min, and the measurement of the absorbance at 734 nm.

The ABTS activity was determined using the following equation (Equation (5)), and results were reported as IC_50_ values, which were estimated by linear regression analysis of the dose–response curve obtained by plotting inhibition percentage against concentration (Figure S5).

(5) ABTS activity%=A0−A1A0×100%,

where *A*0 is the blank absorbance and *A*1 is the sample/standard absorbance.

### 2.8. Evaluation of Acute Toxicity

The acute oral toxicity of the methanolic plant extracts was evaluated in accordance with OECD Guideline 425 [21], using limit doses of 2000 and 5000 mg·kg^−1^ body weight. All individual raw data related to body weight evolution, water intake, and food consumption at doses of 2000 and 5000 mg·kg^−1^ body weight are reported in Supporting Tables S1–S6.

### 2.9. Statistical Analysis

Statistical analysis was carried out using SPSS Software Version 21. Data are presented as mean ± standard deviation. Proximate composition, mineral, and phytochemical analyses were performed in triplicate (*n* = 3). For acute toxicity studies, animals were randomly allocated to experimental groups (*n* = 5 per group). Differences between groups were evaluated using one‐way ANOVA at a 95% confidence level, followed by Tukey′s post hoc test for mean separation. Statistical significance was accepted at *p* < 0.05.

## 3. Results and Discussion

### 3.1. Nutritional Properties

#### 3.1.1. Basic Composition

The basic composition of the tested WEMPs was determined on a dry matter basis and is presented in Table 2.

The moisture content of the studied WEMPs ranged from 6.25% in *Z. lotus* to 10.23% in *O. vulgare* (Table 3). The variation between species may be linked to differences in water‐retention strategies, structural composition, or ecological niches [22]. For instance, species such as *Z. lotus* that grow in drier habitats tend to accumulate less moisture, whereas species like *O. vulgare* may retain slightly more due to differences in leaf texture or growth conditions. From a functional standpoint, moisture is a key factor that influences the shelf life and stability of food products [23]. The low moisture percentage recorded in these WEMPs might be preferable, as it enhances shelf life, contributes to microbiological stability, and thereby improves postharvest preservation. The findings of the present study are comparable to those reported for three WEPs from western Cameroon (8.78%–9.46%) by Ndomou et al. [24]. Comparable results were also reported by Adamu et al. [23] who found that the moisture content ranged from 6.50% in *Haplocarpha schimperi* to 9.77% in *Urtica simensis* in Ethiopia.

**Table 3:** Basic composition of the investigated WEMPs.

Proximate (%, DW)	*Z. lotus*	*O. vulgare*	*D. ambrosioides*
Moisture	6.25 ± 0.022^a^	10.23 ± 0.019^c^	7.15 ± 1.263^b^
Ash	4.81 ± 0.012^a^	8.19 ± 0.014^b^	14.85 ± 0.009^c^
Proteins	6.98 ± 0.009^a^	10.21 ± 0.131^b^	20.43 ± 0.008^c^
Fat	2.88 ± 0.212^c^	2.26 ± 0.414^b^	0.97 ± 0.025^a^
Carbohydrates	85.33 ± 0.211^c^	79.34 ± 0.432^b^	63.75 ± 0.028^a^
Energy (kcal·100 g^−1^)	395.16 ± 2.073^c^	378.54 ± 4.111^b^	345.45 ± 0.252^a^

*Note:* Values are the mean of three independent replicates ± SD. Different superscript letters within each row indicate significant difference (*p* < 0.05).

Ash represents the inorganic residue of plant material and comprises essential dietary components, particularly minerals, including macro and micronutrients that are crucial for normal physiological functions of the body [25]. It consists mainly of oxides and salts, including anions such as sulfates, phosphates, and chlorides, as well as K, Na, Ca, Mg, Fe, and Mn [26]. In the present study, the mean ash content varied from 4.81% in *Z. lotus* fruits to 14.85% in *D. ambrosioides* leaves, as presented in Table 3. These findings were lower than the findings of Mokria et al. [27], who reported ash levels of 8.10%–22.41% in *Ziziphus spina*‐*christi* fruits and *Cordia africana* fruits, respectively. However, our findings were higher than the ash content from 6.37% in *Shapla* stems to 8.68% in *Kalmi Shak* leaves and tender stems [28]. The relatively high ash content, particularly in *D. ambrosioides*, underscores the potential of these WEMPs as important sources of dietary minerals.

Dietary proteins are essential macromolecules that contribute to the growth, maintenance, and repair of body tissues and play an essential role in enzymatic activity, hormone regulation, immune defense, and cellular transport [29]. The protein content of the studied WEMPs ranged from 6.98% in *Z. lotus* to 20.43% in *D. ambrosioides* (Table 3). These results are in line with those reported by Satter et al. [28], who reported a similar protein range. In contrast, a study conducted in northeastern Ethiopia reported protein contents ranging from 13.10% to 33.63% [23], which are greater than those recorded in this study. Based on the protein content data, WEMPs such as *D. ambrosioides* with the highest protein levels can be considered excellent sources of dietary protein, supporting their inclusion in human diets and highlighting their potential role in addressing protein deficiencies, especially in regions where animal protein availability is restricted. However, it should be noted that protein content alone does not fully reflect nutritional quality, and factors such as protein digestibility and amino acid composition should be taken into consideration when evaluating the relative potential of WEMPs as alternative protein food sources.

The mean result of the fat content of WEMPs was between 0.97% and 2.88%. *D. ambrosioides* leaves had the lowest value while *Z. lotus* fruits had the highest value. Values are provided in Table 3. These fat content values were lower than those reported in previous studies conducted in Cameroon [24], Pakistan [30], and Thailand [31]. According to the classification proposed by Trattner and Elsweiler [32], all the WEMPs studied are categorized as low‐fat foods (≤ 3.0 g·100 g^−1^). From a nutritional point of view, the low fat content of these species may be advantageous, as diets characterized by low fat intake, particularly low saturated fat, have been associated with a reduced risk of cardiovascular diseases and obesity [33]. However, the health implications of dietary fats are largely determined by fatty acid composition, especially the balance between saturated and unsaturated fatty acids [34].

Carbohydrates are among the main nutrients in the human diet. Their levels in plants are an important indicator of nutritional and caloric value [35]. As detailed in Table 3, the highest carbohydrate content was observed in *Z. lotus* (85.33%), followed by *O. vulgare* (79.34%) and *D. ambrosioides* (63.75%). The present results were higher than the report by Adamu et al. [23] in the leaves of *Erucastrum arabicum* (30.11%) and *Amaranthus hybridus (*66.25%). Our results were also higher than the finding of Suksathan et al. [31], who reported from 55.44% in *Viburnum inopinatum* to 69.12% in *Glycyrrhiza glabra* in Thailand. It has been reported that the type of carbohydrates is more important in the diet than consuming high or low amounts [36]. Moreover, the total carbohydrate content of wild plants is influenced by plant species, stage of maturity, moisture and fiber contents, and ecological conditions [36].

The energy value of the studied WEMPs ranged from 345.45 to 395.16 kcal·100 g^−1^, as presented in Table 3. The highest caloric value was observed in the fruits of *Z. lotus*, while the lowest value was recorded in the leaves of *D. ambrosioides*. These results are consistent with findings from previous studies conducted in Bangladesh [28], southern Ethiopia [33], and northeastern Ethiopia [23]. However, the current study results were slightly higher than the results reported for wild edible plants in Pakistan, which ranged from 240.3 kcal·100 g^−1^ in *Portulaca oleracea* to 299.59 kcal·100 g^−1^ in *Rumex patientia* [30]. These observations suggest that WEMPs could play a significant role in meeting dietary energy requirements.

#### 3.1.2. Mineral Composition

The mineral composition of the WEMP powders, including Mg, Ca, Na, K, Zn, Fe, Mn, and Cu, classified into macro‐ and microelements, is presented in Table 4. Significant differences (*p* < 0.05) were observed among the studied species for all analyzed minerals.

**Table 4:** Mineral profile of the investigated WEMPs (mg·100 g^−1^ DW).

Extracts	Mg	Ca	Na	K	Zn	Fe	Mn	Cu
*D. ambrosioides*	95.77 ± 0.45^c^	138.43 ± 1.09^b^	197.65 ± 1.82^c^	199.48 ± 0.76^b^	0.68 ± 0.19^c^	4.31 ± 0.03^c^	1.65 ± 0.11^b^	0.23 ± 0.09^c^
*O. vulgare*	65.39 ± 0.95^b^	198.63 ± 0.12^b^	4.97 ± 0.73^b^	182.24 ± 2.03^b^	1.43 ± 0.32^b^	3.65 ± 0.04^b^	1.16 ± 0.19^c^	0.69 ± 0.13^b^
*Z. lotus*	46.76 ± 0.75^a^	167.32 ± 0.24^a^	9.56 ± 0.17^a^	489.87 ± 0.12^a^	1.23 ± 0.16^a^	1.73 ± 0.09^a^	2.02 ± 0.22^a^	1.43 ± 0.07^a^

*Note:* Values are the mean of three independent replicates ± SD. Different superscript letters within each column indicate significant difference (*p* < 0.05).

The Mg concentration in the studied WEMPs (Table 4) was highest in *D. ambrosioides* (95.77 mg·100 g^−1^) and lowest in *Z. lotus* (46.76 mg·100 g^−1^). These results are comparable to those reported for indigenous wild edible plants traditionally consumed in Malaysia [37] but higher than the values reported for WEPs from northeastern Ethiopia, which ranged from 56.65 to 72.79 mg·100 g^−1^ [23]. Mg is an essential macroelement required for protein synthesis, DNA and RNA synthesis, glucose metabolism, and proper nerve function [38]. Diets dominated by refined foods and low in whole grains and green vegetables are commonly associated with Mg deficiency [39]. Mg depletion can impair antioxidant defense mechanisms, leading to increased reactive oxygen species production, as Mg acts as a cofactor for several antioxidant enzymes [39]. Therefore, the combined richness in Mg and phenolic compounds in WEMPs may contribute to antioxidant potential.

Ca is required for bone and tooth development, muscle function, nerve transmission, and detoxification processes [40]. In the present study, Ca levels in the analyzed WEPs (Table 4) were lower than those reported for species from Spain, where concentrations ranged from 16 mg·100 g^−1^ in the basal leaves of *Taraxacum obovatum* to 472 mg·100 g^−1^ in the leaves of *Chondrilla juncea* [41]. In contrast, the Ca levels recorded here were higher than those documented in Ethiopian and Pakistani species, which ranged from 44.35 to 60.14 mg·100 g^−1^ in *Erucastrum arabicum* and *Urtica simensis* and from 5.30 to 11.63 mg·100 g^−1^ in *Portulaca oleracea* and *Amaranthus thunbergii*, respectively [23, 30]. Such variability in Ca content likely reflects species‐specific accumulation capacity, soil mineral composition, and environmental growing conditions. Although the observed Ca levels suggest a moderate dietary contribution, their nutritional relevance may be limited by bioavailability and the presence of antinutritional factors. Thus, these WEMPs are better regarded as complementary sources of Ca.

The highest K content was recorded in *Z. lotus* fruits (489.87 mg·100 g^−1^), while the lowest was found in *O. vulgare* leaves (182.24 mg·100 g^−1^) (Table 4). In contrast, the highest Na content was found in *D. ambrosioides* (197.65 mg·100 g^−1^), while the lowest was found in *O. vulgare* (4.97 mg·100 g^−1^). The physiological functions of K are closely interrelated with Na, and increased K intake combined with reduced Na intake has been shown to lower blood pressure and reduce cardiovascular risk [42, 43]. The Na/K ratio is considered an indicator of dietary quality, with values below 1 considered beneficial for mitigating Na‐related risks [37]. In this study, all three WEMPs present Na/K ratios lower than 1, supporting their potential as suitable plants for low‐Na diets.

The Zn content of the analyzed WEPs ranged from 0.68 mg·100 g^−1^ in the leaves of *D. ambrosioides* to 1.43 mg·100 g^−1^ in the leaves of *O. vulgare*, as presented in Table 4. The Zn levels of these WEPs were higher than the levels reported for certain wild edible plants in Pakistan, where concentrations ranged from 0.07 mg·100 g^−1^ in the aerial parts of *Portulaca oleracea* to 0.69 mg·100 g^−1^ in *Amaranthus thunbergii* [30]. Zn is essential for enzymatic functions, growth, immune response, reproduction, and cardiovascular health [28]. Zn deficiency is associated with complications during pregnancy and childbirth, reduced birth weight, delayed growth, diminished appetite, and general fatigue and remains a major public health concern in developing countries [1]. Despite its essential physiological role, the relatively low Zn levels recorded suggest a limited contribution of these WEMPs to dietary Zn intake.

The Fe content varied significantly (*p* < 0.05) between the three WEMPs examined in Table 4. The highest and lowest Fe content was discovered with *D. ambrosioides* (4.31 mg·100 g^−1^) and *Z. lotus* (1.73 mg·100 g^−1^), respectively. The Fe content in these species was higher than that in previous finding [37], who reported from 0.7 mg·100 g^−1^ in *Strobilanthes crispa* Blume leaves to 2.8 mg·100 g^−1^ in *Sauropus androgynous* (L.) Merr Young shoots in Malaysia. Furthermore, the Fe values recorded in the present study were higher than in commonly consumed vegetables such as spinach (1.05 mg·100 g^−1^), lettuce (0.95 mg·100 g^−1^), and broccoli (0.69 mg·100 g^−1^), which are considered good sources of dietary Fe. The RDA for Fe is 8 mg/day for adult men and postmenopausal women and 18 mg/day for women of reproductive age. Consuming a 200‐g portion of *D. ambrosioides* leaves could provide 80.25% of the RDA for adult men and postmenopausal women and 35.66% for women of reproductive age. However, it is well established that the absorption of Fe of plant origin is strongly influenced by the presence of some Fe‐binding substances, particularly phytates, which can form insoluble complexes and reduce Fe availability. Hallberg et al. [44] recommended a phytate acid/Fe ratio lower than 1 as indicative of favorable Fe absorption. The phytate content of *D. ambrosioides* has been previously reported [45], which allowed the estimation of the phytate/Fe molar ratio. The calculated value (59.4), largely exceeding the recommended threshold for adequate Fe absorption indicates a very low Fe availability. This highlights the importance of considering antinutritional factors when evaluating the nutritional quality of WEMPs.

Mn is an important trace mineral involved in several key physiological processes, including cellular homeostasis, energy metabolism, immune and reproductive systems, antioxidant function, bone formation, neurotransmitter production, and detoxification [46]. Mn levels varied from 1.16 to 2.02 mg·100 g^−1^ in samples of *O. vulgare* and *Z. lotus*, respectively (Table 4). These figures were lower than the results reported for WEPs from Ethiopia and Pakistan, which varied from 7.99 to 19.08 mg·100 g^−1^ and 0.86 to 1.91 mg·100 g^−1^, respectively [23, 30]. Nevertheless, this relatively low Mn concentration is unlikely to represent a nutritional limitation, as Mn deficiency is considered rare compared to other trace element deficiencies.

Cu is an essential microelement that functions as a catalytic, structural, and regulatory cofactor in numerous enzymes within the human body. In addition to Fe, it supports good health, helps prevent anemia, and is associated with the functions of Zn and Fe of the body [1]. Our results showed that Cu levels in the species examined in this study (Table 4) were relatively lower than those found in some wild edible plants in Bangladesh, where concentrations ranged from 0.15 mg·100 g^−1^ in the leaves and tender stems of *Corchorus capsularis* to 3.25 mg·100 g^−1^ in the stems and leaves of *Dryopteris filix-mas* [28]. In contrast, these levels were higher than those documented for Moroccan species, which varied between 0.59 mg·100 g^−1^ in *Rubia peregrina* and 0.89 mg·100 g^−1^ in *Lavandula stoechas* [9]. The Cu concentrations recorded in the present study were within the WHO permissible limits for foods (4 mg·100 g^−1^).

### 3.2. Extraction Yield

**Figure 1:**
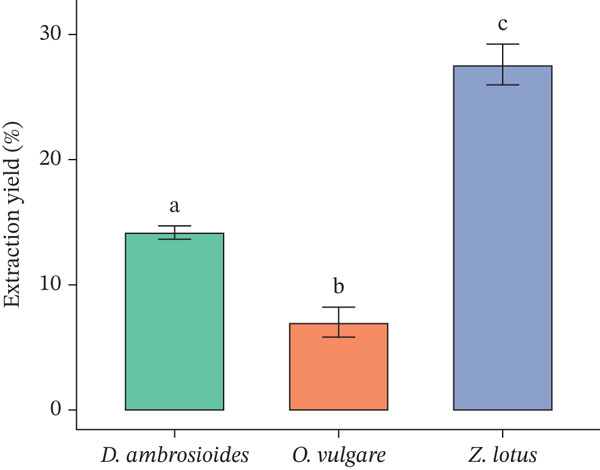
Extraction yield percentage of methanolic plant extracts. Values are the mean of three independent replicates ± SD. Different superscript letters above the bars indicate significant difference (*p* < 0.05).

The percentage yields of plant extracts are shown in Figure 1. The extraction yield of the studied plants ranged from 6.95% to 27.56%. These values do not deviate from the reports of Slimestad et al. [47] and Letaief et al. [48] for *O. vulgare* and *Z. lotus*. However, the extract yield reported by Kandi et al. [49] for *D. ambrosioides* (18%–47%) was higher than our results. These differences could be explained by the solvent, extraction method, and plant part used, which differed from those in our study.

### 3.3. TPC

Polyphenols are bioactive food constituents that are widely recognized for their functional and therapeutic properties in the human body [50]. The TPC of studied wild edible medicinal species is presented in Table 5. The TPC in the methanolic extracts varied significantly (*p* < 0.05), from 64.45 ± 0.15 to 207.30 ± 0.52 mg GAE·g^−1^ extract. The extract from *O. vulgare* leaves exhibited the highest TPC, while the lowest value was observed in the fruit extract of *Z. lotus*. The TPC of *O. vulgare* obtained in our study was comparable to that reported by Ličina et al. [51], which found values ranging from 84.5 ± 0.59 to 235 ± 1.73 mg GAE·g^−1^ of extract. In contrast, our result was higher than that reported in [52], which observed TPC values ranging from 7.27 ± 0.06 to 14.11 ± 0.14 mg GAE·g^−1^ in hydromethanolic extracts of *O. vulgare* leaves and stems. For *D. ambrosioides*, the methanolic extract analyzed in the present study showed a TPC of 116.59 ± 0.19 mg GAE·g^−1^, which was significantly higher than the value reported by Zohra et al. [53], who recorded a TPC of 87.69 ± 1.41 mg GAE·g^−1^ extract. Similarly, the TPC of *Z. lotus* extract in this study (64.45 ± 0.15 mg GAE·g^−1^ extract) exceeded the range reported by Rais et al. [54], which varied from 13.79 ± 0.07 to 30.36 ± 0.30 mg GAE·g^−1^ extract. When expressed on a dry weight basis, the TPC ranged from 14.41 to 17.77 mg GAE·g^−1^ DW among the studied species. Despite exhibiting the highest TPC per gram of extract, *O. vulgare* showed a moderate phenolic content on a dry matter basis due to its lower extraction yield. In contrast, *Z. lotus* displayed a higher TPC per gram of dry matter, which reflects its greater extract yield. These findings indicate that phenolic richness at the extract level does not necessarily correspond to phenolic abundance in the plant matrix. Therefore, expressing TPC on both extract and dry weight bases provides a more accurate comparison of the phenolic potential of WEMPs.

**Table 5:** Total polyphenolic and flavonoid contents of studied WEMPs.

Extracts	TPC (mg GAE·g^−1^ extract)	TPC (mg GAE·g^−1^ DW)	TFC (mg QE·g^−1^ extract)	TFC (mg QE·g^−1^ DW)
*D. ambrosioides*	116.59 ± 0.19^b^	16.44 ± 0.19^b^	21.76 ± 0.34^b^	3.07 ± 0.32^a^
*O. vulgare*	207.30 ± 0.52^c^	14.41 ± 0.51^a^	117.18 ± 0.34^c^	8.14 ± 0.29^c^
*Z. lotus*	64.45 ± 0.15^a^	17.77 ± 0.12^c^	18.84 ± 1.03^a^	5.19 ± 0.14^b^

*Note:* Values are the mean of three independent replicates ± SD. Different superscript letters within each column indicate significant difference (*p* < 0.05). GAE and QE refer to gallic acid and quercetin, respectively.

### 3.4. TFC

Flavonoids are naturally occurring plant compounds that constitute a major subclass of polyphenols. Owing to their potent antioxidant activity, they have been extensively studied for their potential health benefits, including the prevention of cardiovascular diseases, attenuation of inflammatory responses, reduction in cancer risk, and protection of human cells against mutagenic damage [55]. In the present study, the TFC of WEMPs ranged from 18.84 ± 1.03 mg QE·g^−1^ in *Z. lotus* to 117.18 ± 0.34 mg QE·g^−1^ in *O. vulgare*, as reported in Table 5. These findings align with the report by Ličina et al. [51], who found TFCs ranging from 57.1 ± 11.8 to 132 ± 8.3 mg RE·g^−1^ extract in *O. vulgare*. In contrast, a considerably higher TFC of 2242.89 ± 25 mg QE·g^−1^ extract for *Z. lotus* was reported [56] compared to our findings. Similarly, a TFC of 57 ± 1.41 mg QE·g^−1^ extract for *D. ambrosioides* was recorded [53], which also exceeds the value obtained in the present study. The variation in phytochemical composition among WEMPs may be attributed to both intrinsic factors (such as genetic makeup) and extrinsic factors (including geographical origin, climatic conditions, seasonality, harvest time, and the specific plant part analyzed), as well as to the complex interactions among these variables [31].

### 3.5. Antioxidant Activity

Three different in vitro assays, namely, DPPH, ABTS, and FRAP, were employed to evaluate the antioxidant activity of the studied WEMPs, as reliance on a single assay may underestimate or misrepresent the antioxidant potential of complex plant extracts. Overall, Table 6 shows that all studied wild edible plants displayed strong antioxidant activity, although significant differences were observed (*p* < 0.05), ranging from 0.003 to 0.114 mg·mL^−1^ for DPPH, from 0.370 to 1.970 mg·mL^−1^ for ABTS, and from 0.0078 to 0.114 mg·mL^−1^ for FRAP assays. It was found that *O. vulgare* always exhibited the highest antioxidant capacities across the three assays, followed by *D. ambrosioides* and *Z. lotus*. These findings are comparable to the IC_50_ values reported for selected Ugandan wild edible plants, which ranged from 0.013 to 0.064 mg·mL^−1^ against DPPH [57]. Furthermore, the studied wild edible plants exhibited considerably higher antioxidant potential than several commonly consumed fruits and vegetables, such as beans (1.580 mg·mL^−1^ for DPPH and 4.080 mg·mL^−1^ for FRAP), carrot (3.770 mg·mL^−1^ for DPPH and 76.250 mg·mL^−1^ for FRAP), cucumber (7.210 mg·mL^−1^ for DPPH and 60.910 mg·mL^−1^ for FRAP), and tomato (0.960 mg·mL^−1^ for DPPH and 5.220 mg·mL^−1^ for FRAP) [58].

**Table 6:** Antioxidant activity of studied WEMPs.

Extracts	IC_50_ DPPH (mg·mL^−1^)	IC_50_ ABTS (mg mL^−1^)	IC_50_ FRAP (mg·mL^−1^)
*D. ambrosioides*	0.007 ± 0.34^b^	0.430 ± 0.59^b^	0.042 ± 0.24^b^
*O. vulgare*	0.003 ± 1.17^a^	0.370 ± 0.12^a^	0.0078 ± 0.64^a^
*Z. lotus*	0.114 ± 1.89^c^	1.970 ± 0.15^c^	0.114 ± 2.03^c^

*Note:* Values are the mean of three independent replicates ± SD. Different superscript letters within each column indicate significant difference (*p* < 0.05).

Phenolic compounds are well known as key plant constituents responsible for antioxidant activity due to their intrinsic redox properties. These bioactive molecules contribute significantly to the overall antioxidant potential of plant extracts by acting through multiple mechanisms, including free radical scavenging, reducing activity, hydrogen donation, metal ion chelation, and singlet oxygen quenching [59]. Considering this, correlation analysis was performed to examine the relationship between polyphenol composition and antioxidant activities of the selected WEPs (Table 7). The results revealed very strong correlations between TPC and TFC of WEMPs (*r* = 0.942), as well as between FRAP and TPC (*r* = 0.938), DPPH and TPC (*r* = 0.799), ABTS and TPC (*r* = 0.799), all of which were highly significant (*p* < 0.01). Currently, several studies have revealed a significant relation between antioxidant ability and phenolic content [23, 33, 57, 58] and that confirms the findings of the present study.

**Table 7:** Pearson′s correlation analysis of phenolic and flavonoid contents with antioxidant activities.

	Phenolic	Flavonoids	DPPH	ABTS	FRAP
Phenolic	1.000				
Flavonoids	0.942 ^∗∗^	1.000			
DPPH	−0.799 ^∗∗^	−0.549	1.000		
ABTS	−0.799 ^∗∗^	−0.550	1.000	1.000	
FRAP	−0.938 ^∗∗^	−0.766 ^∗^	0.958 ^∗∗^	0.958 ^∗∗^	1.000

^∗^Significant correlation at *p* ≤ 0.05;  ^∗∗^significant correlation at *p* ≤ 0.01.

### 3.6. Acute Toxicity

The acute toxicity of the methanolic extract of WEMPs was assessed according to OECD Guideline 425 [21], using a limit test dose of 5000 mg·kg^−1^ body weight. Throughout the 14‐day acute toxicity observation period, no noticeable signs or symptoms of toxicity, such as loss of reflexes, drowsiness, edema, tremors, diarrhea, and excessive urination or salivation, were observed. In addition, no mortality was detected in any of the dosed animals at either 2000 or 5000 mg·kg^−1^ doses (Table 8). Based on the Globally Harmonized System (GHS) classification criteria, *D. ambrosioides*, *O. vulgare*, and *Z. lotus* extracts can be classified under Category 5 (plants with a median lethal dose [LD50] exceeding 5000 mg·kg^−1^).

**Table 8:** Monitoring of appearance, behavior, and living habits after oral administration of plant extracts (2000 and 5000 mg·kg^−1^).

Clinical signs	1 h			2 h			3 h			4 h			Day 2 to Day 14		
	*DA*	*OV*	*ZL*	*DA*	*OV*	*ZL*	*DA*	*OV*	*ZL*	*DA*	*OV*	*ZL*	*DA*	*OV*	*ZL*
LR	−	−	−	−	−	−	−	−	−	−	−	−	−	−	−
Locomotion	−	−	−	−	−	−	−	−	−	−	−	−	−	−	−
Drowsiness	−	−	−	−	−	−	−	−	−	−	−	−	−	−	−
Edema	−	−	−	−	−	−	−	−	−	−	−	−	−	−	−
Urination	−	−	−	−	−	−	−	−	−	−	−	−	−	−	−
Salivation	−	−	−	−	−	−	−	−	−	−	−	−	−	−	−
Diarrhea	−	−	−	−	−	−	−	−	−	−	−	−	−	−	−
IRS	−	−	−	−	−	−	−	−	−	−	−	−	−	−	−
ECT	+	+	+	+	+	+	+	+	+	+	+	+	+	+	+
Tremor	−	−	−	−	−	−	−	−	−	−	−	−	−	−	−
Mortality	−	−	−	−	−	−	−	−	−	−	−	−	−	−	−

Abbreviations: *DA*, *Dysphania ambrosioides*; ECT, eye closure at touch; IRS, increased respiration rate; LR, loss of reflux; *OV*, *Origanum vulgare*; *ZL*, *Ziziphus lotus.*

Body weight is an indicator of the animal′s overall health, and weight loss is frequently one of the first signs of adverse effects. The body weights of the control and dosed groups are presented in Figure 2. There were no statistically significant changes (*p* > 0.05) in body weight gain between the control and treated groups throughout the experimental period, which indicates that the extracts were not acutely toxic.

Figure 2Effect of (a) *D. ambrosioides*, (b) *O. vulgare*, and (c) *Z. lotus* extracts on body weight, in experimental rats during acute toxicity studies. Data are presented as mean ± SD (*n* = 5 per group). No significant differences (*p* > 0.05) were observed compared with the control group.

**Figure:**
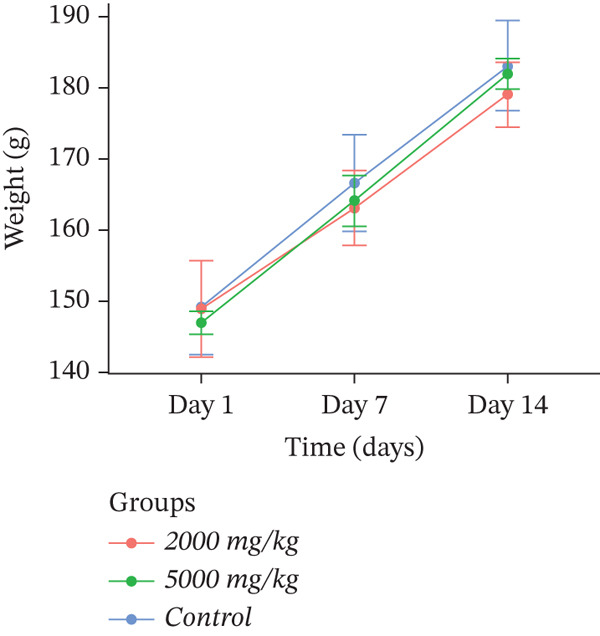
(a)

**Figure:**
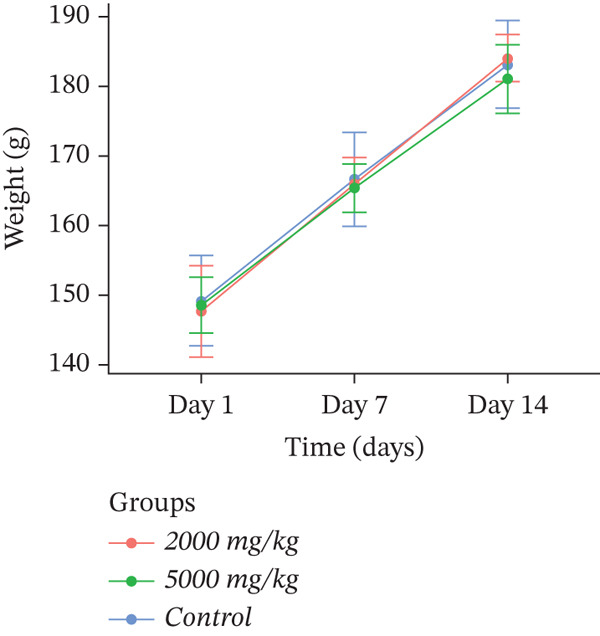
(b)

**Figure:**
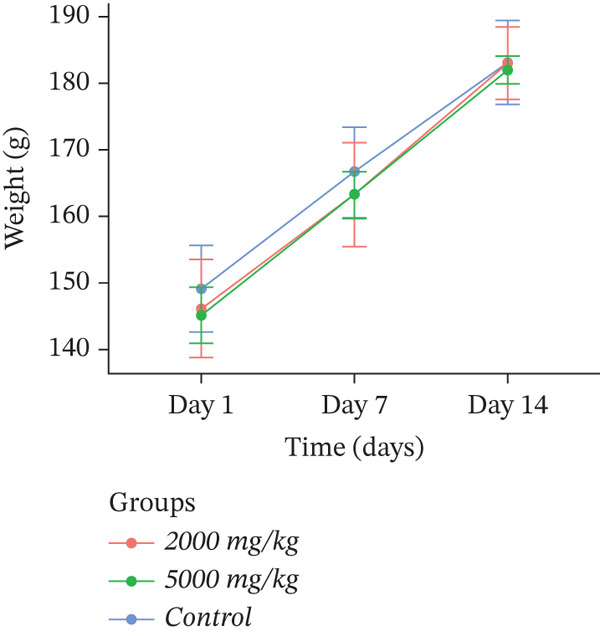
(c)

Furthermore, there were no statistically significant differences (*p* > 0.05) in the food and water intake between the extract‐treated and control groups (Figure 3), which supports the absence of oral acute toxicity effects under the experimental conditions. The present study was restricted to clinical observations, body weight changes, and mortality assessment, in line with OECD Guideline 425, which does not mandate biochemical or histopathological analyses for acute toxicity screening. However, the absence of serum biochemical parameters and histopathological examination represents a limitation of this study, and further subacute or chronic toxicity studies are required to fully establish the safety profile of these extracts.

Figure 3Effect of WEMP extracts on (a) food intake and (b) water intake, in experimental rats during acute toxicity studies. Data are presented as mean ± SD (*n* = 5 per group). The same superscript letters above the bars indicate no significant difference (*p* > 0.05).

**Figure:**
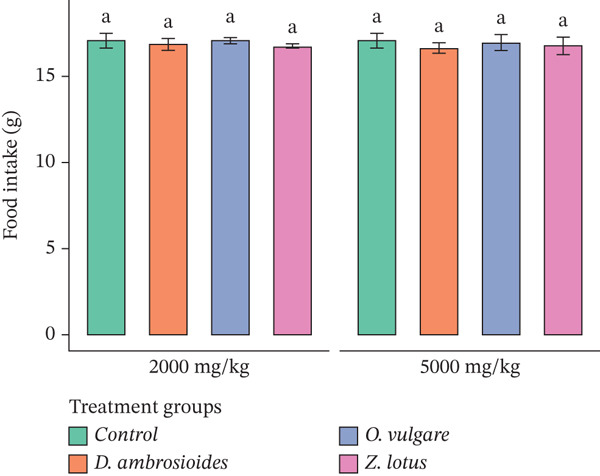
(a)

**Figure:**
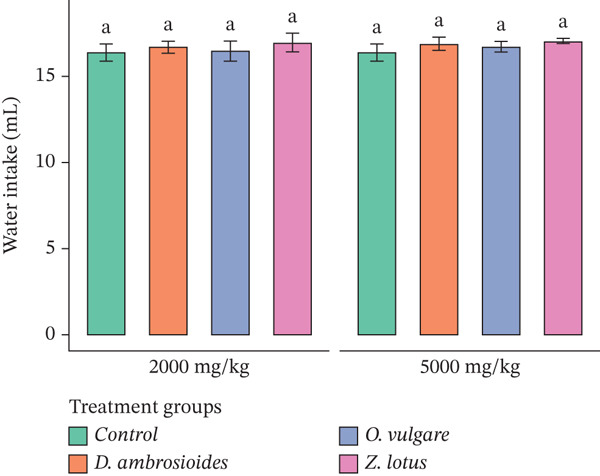
(b)

The absence of acute toxicity effects observed for *Z. lotus* and *O. vulgare* is in agreement with previously published data [60, 61]. In contrast, discrepancies reported for *D. ambrosioides*, including an LD_50_ of 5000 mg· kg^−1^ body weight for hydroethanolic flower extracts reported by Kandsi et al. [62], may be attributed to differences in extraction solvent, plant part used, and resulting phytochemical profiles.

## 4. Conclusion

Based on the above findings, the WEMPs undertaken in the current study can be considered as a potential source of macronutrients, micronutrients, and bioactive molecules with significant antioxidant properties beneficial to human health. Moreover, acute toxicity assessment revealed no adverse effects under the experimental conditions. Understanding their antioxidant and nutraceutical potential may stimulate further research into their use in the nutraceutical industry and support future pharmacological investigations.

## Author Contributions

Conceptualization, Abdelghani Aboukhalaf and Adil Kalili; methodology, Abdelghani Aboukhalaf, Youssef Lahlou, and Jamila El Biyad; formal analysis, Abdelghani Aboukhalaf and Rekia Belahsen; investigation, Youssef Lahlou and Houda El Yacoubi; resources, Abdelghani Aboukhalaf and Rekia Belahsen; writing—original draft preparation, Abdelghani Aboukhalaf, Houda El Yacoubi, and Rekia Belahsen; writing—review and editing, Abdelghani Aboukhalaf, Atmane Rochdi, and Rekia Belahsen; supervision, Rekia Belahsen.

## Funding

No funding was received for this manuscript.

## Disclosure

All authors have read and agreed to the published version of the manuscript.

## Ethics Statement

The current study was approved by the Animal Ethics Review Committee of the Faculty of Sciences of Kenitra, Morocco, under Ethical Authorization Number 11/2024/NRSDL. All experimental procedures were conducted in compliance with international standards for laboratory animal care.

## Consent

The authors have nothing to report.

## Conflicts of Interest

The authors declare no conflicts of interest.

## Supporting information


**Supporting Information** Additional supporting information can be found online in the Supporting Information section. Figures S1–S5: Calibration curves (gallic acid and quercetin) and dose–response curves of *O. vulgare*, *D. ambrosioides*, and *Z. lotus* extracts obtained from DPPH, FRAP, and ABTS antioxidant assays. Tables S1–S6: Individual raw data for body weight evolution, water intake, and food consumption of rats treated with plant extracts at doses of 2000 and 5000.

## Data Availability

The data used to support the findings of this study are available from the corresponding author upon request.
