# Supplementary material for: Nutritional, Bioactive, Antioxidant, and Safety Assessment of Some Wild Edible Medicinal Plants Commonly Consumed in Morocco
Source: Int J Food Sci. 2026 Apr 24;2026:5125567. doi: 10.1155/ijfo/5125567 (PMC13109073; doi:10.1155/ijfo/5125567)
Supplement: Supplementary file 1 — Supporting Information Additional supporting information can be found online in the Supporting Information section. Figures S1–S5: Calibration curves (gallic acid and quercetin) and dose–response curves of O. vulgare, D. ambrosioides, and Z. lotus extracts obtained from DPPH, FRAP, and ABTS antioxidant assays. Tables S1–S6: Individual raw data for body weight evolution, water intake, and food consumption of rats treated with plant extracts at doses of 2000 and 5000. [file IJFO-2026-5125567-s001.docx]

**Figure S1:** Standard gallic acid calibration curve.

**Figure S2:** Standard quercetin calibration curve.

**Figure S3:** Dose-response curves of *O. vulgare* (A), *D. ambrosioides* (B) and *Z. lotus* (C) extracts in the DPPH assay.

**Figure S4:** Dose-response curves of *O. vulgare* (A), *D. ambrosioides* (B) and *Z. lotus* (C) extracts in the FRAP assay.

**Figure S5:** Dose-response curves of *O. vulgare* (A), *D. ambrosioides* (B) and *Z. lotus* (C) extracts in the ABTS assay.

**Table S1**. Evolution of body weight (g) in rats at 2000 mg·kg⁻¹ body weight (BW).

| **Group** | **Rat ID** | **DAY 1** | **DAY 7** | **DAY 14** |
| --- | --- | --- | --- | --- |
| Control | R1 | 146,9 | 166 | 185,5 |
| Control | R2 | 155 | 172,6 | 189,4 |
| Control | R3 | 141 | 157,7 | 174,4 |
| Control | R4 | 146,2 | 162,9 | 178,7 |
| Control | R5 | 156,6 | 173,8 | 187,4 |
| *D. ambrosioides* | R1 | 146,6 | 161,7 | 175,8 |
| *D. ambrosioides* | R2 | 144 | 161,4 | 177,3 |
| *D. ambrosioides* | R3 | 160,3 | 171,3 | 186,7 |
| *D. ambrosioides* | R4 | 150 | 164,1 | 179,4 |
| *D. ambrosioides* | R5 | 144,1 | 156,9 | 175,9 |
| *O. vulgare* | R1 | 153 | 168,6 | 184,8 |
| *O. vulgare* | R2 | 156,3 | 168,7 | 186,3 |
| *O. vulgare* | R3 | 144,2 | 168,8 | 187,9 |
| *O. vulgare* | R4 | 141,2 | 160,9 | 179,7 |
| *O. vulgare* | R5 | 143,6 | 162,1 | 181,5 |
| *Z. lotus* | R1 | 141,1 | 155,4 | 178,6 |
| *Z. lotus* | R2 | 151,4 | 167,5 | 187,4 |
| *Z. lotus* | R3 | 141,3 | 158,4 | 178,9 |
| *Z. lotus* | R4 | 140,5 | 160,1 | 179,8 |
| *Z. lotus* | R5 | 156,4 | 174,8 | 190,3 |

**Table S2**. Evolution of body weight (g) in rats at 5000 mg·kg⁻¹ body weight (BW).

| **Group** | **Rat ID** | **DAY 1** | **DAY 7** | **DAY 14** |
| --- | --- | --- | --- | --- |
| Control | R1 | 152,5 | 169,2 | 186,4 |
| Control | R2 | 153,8 | 172,7 | 186,2 |
| Control | R3 | 143,6 | 159,6 | 181,4 |
| Control | R4 | 147,8 | 166,2 | 183 |
| Control | R5 | 147,4 | 164,9 | 178,5 |
| *D. ambrosioides* | R1 | 149,1 | 167,8 | 184,7 |
| *D. ambrosioides* | R2 | 147 | 166,5 | 183,4 |
| *D. ambrosioides* | R3 | 145,7 | 161,2 | 181,8 |
| *D. ambrosioides* | R4 | 145,3 | 159,4 | 180,7 |
| *D. ambrosioides* | R5 | 148 | 165,4 | 179,4 |
| *O. vulgare* | R1 | 155,2 | 171,2 | 189,6 |
| *O. vulgare* | R2 | 145,2 | 165,5 | 180,2 |
| *O. vulgare* | R3 | 148,3 | 164,1 | 180,3 |
| *O. vulgare* | R4 | 145,5 | 162,6 | 177,1 |
| *O. vulgare* | R5 | 148,2 | 163,2 | 178 |
| *Z. lotus* | R1 | 151,4 | 167,9 | 185,3 |
| *Z. lotus* | R2 | 141,4 | 161,1 | 179,4 |
| *Z. lotus* | R3 | 141,2 | 158,8 | 181,1 |
| *Z. lotus* | R4 | 146,3 | 165 | 182,1 |
| *Z. lotus* | R5 | 145 | 163 | 182,2 |

**Table S3:** Water intake of rats on days 1, 7, and 14 after oral administration of plant extracts at 2000 mg·kg⁻¹ BW.

| **Group** | **Rat ID** | **DAY 1** | **DAY 7** | **DAY 14** |
| --- | --- | --- | --- | --- |
| Control | R1 | 15,6 | 17,4 | 17,1 |
| Control | R2 | 14,9 | 16,2 | 17,3 |
| Control | R3 | 14,7 | 16 | 16,8 |
| Control | R4 | 16,1 | 15,9 | 16,2 |
| Control | R5 | 16,7 | 17,7 | 16,8 |
| *D. ambrosioides* | R1 | 17,3 | 16,2 | 17,8 |
| *D. ambrosioides* | R2 | 16,3 | 17,3 | 17,5 |
| *D. ambrosioides* | R3 | 15,4 | 17,6 | 16,1 |
| *D. ambrosioides* | R4 | 15,1 | 17,1 | 16,9 |
| *D. ambrosioides* | R5 | 14,9 | 17,8 | 17 |
| *O. vulgare* | R1 | 16,3 | 15,6 | 17,4 |
| *O. vulgare* | R2 | 17,1 | 17,9 | 17,2 |
| *O. vulgare* | R3 | 15,1 | 16,2 | 18,1 |
| *O. vulgare* | R4 | 15 | 16,1 | 17,1 |
| *O. vulgare* | R5 | 14,9 | 17 | 15,8 |
| *Z. lotus* | R1 | 18,5 | 17,5 | 17,8 |
| *Z. lotus* | R2 | 17,1 | 16,2 | 16,5 |
| *Z. lotus* | R3 | 16,2 | 17,3 | 16,7 |
| *Z. lotus* | R4 | 16,6 | 16,8 | 17 |
| *Z. lotus* | R5 | 16,4 | 16,7 | 16,9 |

**Table S4:** Water intake (ml) of rats on days 1, 7, and 14 after oral administration of plant extracts at 5000 mg·kg⁻¹ BW.

| **Group** | **Rat ID** | **DAY 1** | **DAY 7** | **DAY 14** |
| --- | --- | --- | --- | --- |
| Control | R1 | 15,6 | 17,4 | 17,1 |
| Control | R2 | 14,9 | 16,2 | 17,3 |
| Control | R3 | 14,7 | 16 | 16,8 |
| Control | R4 | 16,1 | 15,9 | 16,2 |
| Control | R5 | 16,7 | 17,7 | 16,8 |
| *D. ambrosioides* | R1 | 18,1 | 17,1 | 17,3 |
| *D. ambrosioides* | R2 | 17,3 | 16,6 | 16,4 |
| *D. ambrosioides* | R3 | 17,4 | 16,1 | 16,9 |
| *D. ambrosioides* | R4 | 16,8 | 16,7 | 16,2 |
| *D. ambrosioides* | R5 | 15,6 | 16,1 | 17,9 |
| *O. vulgare* | R1 | 15,9 | 16,1 | 16,8 |
| *O. vulgare* | R2 | 17,3 | 16,8 | 15,9 |
| *O. vulgare* | R3 | 16,4 | 16,2 | 17,7 |
| *O. vulgare* | R4 | 16,2 | 17,4 | 17,7 |
| *O. vulgare* | R5 | 16,8 | 16,6 | 16,4 |
| *Z. lotus* | R1 | 16,1 | 18,2 | 17,4 |
| *Z. lotus* | R2 | 16,9 | 16,8 | 16,9 |
| *Z. lotus* | R3 | 18,2 | 16,3 | 16,2 |
| *Z. lotus* | R4 | 17,9 | 15,5 | 18,1 |
| *Z. lotus* | R5 | 16,9 | 17,2 | 17,1 |

**Table S5:** Food intake (g) of rats on days 1, 7, and 14 after oral administration of plant extracts at 2000 mg·kg⁻¹ BW.

| **Group** | **Rat ID** | **DAY 1** | **DAY 7** | **DAY 14** |
| --- | --- | --- | --- | --- |
| Control | R1 | 17,4 | 18,2 | 17,8 |
| Control | R2 | 16,8 | 17,1 | 16,7 |
| Control | R3 | 15,9 | 17 | 17,8 |
| Control | R4 | 16,9 | 16,4 | 16,9 |
| Control | R5 | 17,7 | 16,1 | 16,8 |
| *D. ambrosioides* | R1 | 16,3 | 17,8 | 17,1 |
| *D. ambrosioides* | R2 | 15,5 | 17,8 | 16,9 |
| *D. ambrosioides* | R3 | 15,1 | 16,9 | 16,8 |
| *D. ambrosioides* | R4 | 16,3 | 18,1 | 17,1 |
| *D. ambrosioides* | R5 | 16,9 | 17 | 16,6 |
| *O. vulgare* | R1 | 15,8 | 16,8 | 17 |
| *O. vulgare* | R2 | 16,9 | 17,7 | 16,1 |
| *O. vulgare* | R3 | 16 | 17,4 | 17,4 |
| *O. vulgare* | R4 | 16,7 | 16,9 | 16,2 |
| *O. vulgare* | R5 | 16,4 | 17,5 | 17,1 |
| *Z. lotus* | R1 | 15,5 | 16,3 | 18 |
| *Z. lotus* | R2 | 16,6 | 16,8 | 16,2 |
| *Z. lotus* | R3 | 17,1 | 16,8 | 16,1 |
| *Z. lotus* | R4 | 15,9 | 17,8 | 16,9 |
| *Z. lotus* | R5 | 16,6 | 16,4 | 17,2 |

**Table S6:** Food intake (g) of rats on days 1, 7, and 14 after oral administration of plant extracts at 5000 mg·kg⁻¹ BW.

| **Group** | **Rat ID** | **DAY 1** | **DAY 7** | **DAY 14** |
| --- | --- | --- | --- | --- |
| Control | R1 | 17,4 | 18,2 | 17,8 |
| Control | R2 | 16,8 | 17,1 | 16,7 |
| Control | R3 | 15,9 | 17 | 17,8 |
| Control | R4 | 16,9 | 16,4 | 16,9 |
| Control | R5 | 17,7 | 16,1 | 16,8 |
| *D. ambrosioides* | R1 | 15,8 | 16,4 | 17,6 |
| *D. ambrosioides* | R2 | 16,2 | 16,9 | 17,3 |
| *D. ambrosioides* | R3 | 16 | 16,3 | 17,6 |
| *D. ambrosioides* | R4 | 15,9 | 16,7 | 17,9 |
| *D. ambrosioides* | R5 | 15,7 | 16,3 | 16,3 |
| *O. vulgare* | R1 | 15,4 | 17,8 | 17,5 |
| *O. vulgare* | R2 | 15,3 | 16,9 | 16,3 |
| *O. vulgare* | R3 | 16,6 | 16,9 | 17,2 |
| *O. vulgare* | R4 | 16,9 | 17,2 | 18,3 |
| *O. vulgare* | R5 | 17 | 17,1 | 17,1 |
| *Z. lotus* | R1 | 16 | 17,2 | 17,6 |
| *Z. lotus* | R2 | 16,6 | 18,1 | 17,6 |
| *Z. lotus* | R3 | 15,8 | 16,1 | 17,8 |
| *Z. lotus* | R4 | 15,3 | 17,1 | 16,2 |
| *Z. lotus* | R5 | 16,3 | 16,2 | 16,7 |
